# Respiratory microbiome and epithelial interactions shape immunity in the lungs

**DOI:** 10.1111/imm.13195

**Published:** 2020-04-14

**Authors:** Rachele Invernizzi, Clare M. Lloyd, Philip L. Molyneaux

**Affiliations:** ^1^ Inflammation, Repair and Development Section National Heart and Lung Institute Imperial College London UK; ^2^ Department of Respiratory Medicine Interstitial Lung Disease Unit Royal Brompton Hospital London UK

**Keywords:** immunity, lungs, mucosal immunology, respiratory epithelium, respiratory microbiome

## Abstract

The airway epithelium represents a physical barrier to the external environment acting as the first line of defence against potentially harmful environmental stimuli including microbes and allergens. However, lung epithelial cells are increasingly recognized as active effectors of microbial defence, contributing to both innate and adaptive immune function in the lower respiratory tract. These cells express an ample repertoire of pattern recognition receptors with specificity for conserved microbial and host motifs. Modern molecular techniques have uncovered the complexity of the lower respiratory tract microbiome. The interaction between the microbiota and the airway epithelium is key to understanding how stable immune homeostasis is maintained. Loss of epithelial integrity following exposure to infection can result in the onset of inflammation in susceptible individuals and may culminate in lung disease. Here we discuss the current knowledge regarding the molecular and cellular mechanisms by which the pulmonary epithelium interacts with the lung microbiome in shaping immunity in the lung. Specifically, we focus on the interactions between the lung microbiome and the cells of the conducting airways in modulating immune cell regulation, and how defects in barrier structure and function may culminate in lung disease. Understanding these interactions is fundamental in the search for more effective therapies for respiratory diseases.

## Graphical abstract

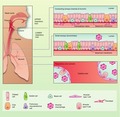

**Abbreviations**
AEC1: alveolar epithelial type 1 cells
AEC2: alveolar epithelial type 2 cells
BAL: bronchoalveolar lavage
CF: cystic fibrosis
CLRs: C‐type lectin receptors
COPD: chronic obstructive pulmonary disease
DKK: Dikkopf
EGFR: epidermal growth factor receptor
GORD: gastro‐oesophageal reflux disease
HHV: human herpes virus
ICAM‐1: intracellular adhesion molecule 1
IPF: idiopathic pulmonary fibrosis
KRM: Kreme
Man‐6‐P: mannose‐6‐phosphate
NLRs: NOD‐like receptors
NOD: nucleotide‐binding and oligomerization domain
PAF: platelet‐activating factor
PAMPs: pathogen‐associated molecular patterns
PARs: protease‐activated receptors
PRR: pattern recognition receptors
RLRs: retinoic acid‐inducible gene‐I‐like receptors
RSV: respiratory syncytial virus
SCFA: short‐chain fatty acid
TARC: thymus‐ and activation‐regulated chemokine
TEER: transepithelial electrical resistance
Th2: T helper 2
TIR: toll/IL‐1 receptor
TLRs: toll‐like receptors
TRIF: TIR‐domain‐containing adapter‐inducing interferon‐β
TSLP: thymic stromal lymphopoietin

## The epithelial barrier

**Figure 1:**
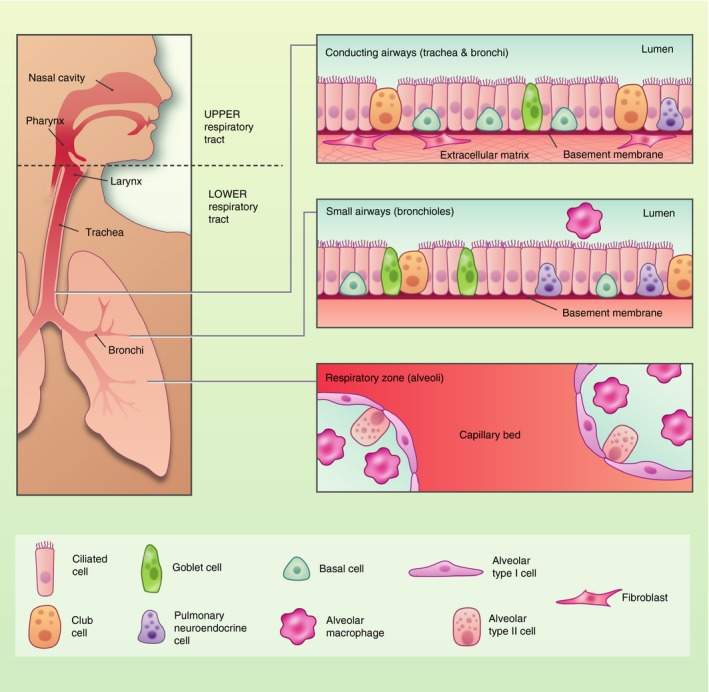
Composition of the respiratory epithelium in health. The human respiratory tract is divided into the upper respiratory tract including the nasal cavity, pharynx, larynx, and the lower respiratory tract that comprises the conducting airways (trachea and bronchi), the small airways (bronchioles) and the respiratory zone (the alveoli). The trachea and large airways are composed of ciliated cells, mucus‐secreting goblet cells, neuroendocrine cells and undifferentiated basal cells. Stromal cells including fibroblasts provide extracellular matrix that modulate airway epithelial cell turnover. The alveoli are comprised of alveolar macrophages and alveolar type I and type II cells.

The human respiratory tract is the main portal of entry for the continuous immigration and elimination of a vast array of airborne microorganisms and particles, including viruses, bacteria and fungi. The lungs are continually exposed to a multitude of microorganisms, some of which are able to persist and colonize the respiratory tract. These microbes that reside in the lower airways constitute the lung microbiome, which is distinct in composition from the microbiota observed in the oral and nasal cavities. The surface of the lungs presents a continuous layer of epithelial cells that constitute a physical and biological barrier for inhaled substances and pathogens. The specialized respiratory epithelium is required for maintenance of immune homeostasis, and epithelial dysfunction is involved in the development of many inflammatory disorders of the airways and lungs.^1^ The respiratory tract is a complex organ system, and its primary function is the exchange of oxygen and carbon dioxide. It is divided into the upper respiratory tract including the nasal cavity, pharynx, larynx, and the lower respiratory tract that comprises the conducting airways (trachea and bronchi), the small airways (bronchioles) and the respiratory zone (the alveoli). The adult human airways have a surface area of approximately 70 m^2^, which harbours a vast range of bacterial communities, with the highest bacterial load observed in the upper respiratory tract.^2^ The respiratory tract spans different anatomical sites, and the types of epithelial cells that comprise these sites are varied both in composition and in structure, reflecting the distinct functions of the airway epithelial cells lining the conducting airways and the alveolar regions of the lungs (Fig. 1).

In the lower respiratory tract, the respiratory epithelium provides a highly effective barrier acting as the first line of defence against potentially harmful environmental stimuli including microbes and allergens.^1^ The pseudostratified epithelium of the conducting airways is primarily comprised of multi‐ciliated cells, mucus‐secreting goblet cells, neuroendocrine cells and basal cells, which secrete surfactant.^1^ It constitutes the first site of interaction with inhaled environmental compounds, and is designed to facilitate effective mucociliary clearance of particles and microbes.^3^ Multi‐ciliated cells have cilia on the apical surface that beat co‐ordinately to shuttle inhaled particulates and mucus out of the airways whilst goblet and secretory cells trap inhaled particulates and microorganisms. Basal cells are progenitor cells that act as resident stem cells for the trachea and proximal airways. These cells are capable of self‐renewal as well as repopulating the pseudostratified epithelium during homeostasis and after injury.^4^ The tracheal region and large airways harbour stromal cells such as interstitial fibroblasts that aid in the regulation of the regenerative response of the airway epithelium after injury.^5^ In contrast, the alveolar surfaces in the peripheral lung are lined by flat alveolar epithelial type 1 cells (AEC1) that form a continuous cell layer and are specialized in gas exchange, while cuboidal alveolar epithelial type 2 cells (AEC2) act as progenitor cells and secrete pulmonary surfactant, which reduces surface tension in order to prevent alveolar collapse during respiration.^6^, ^7^ A common feature shared by these cell types is the presence of intracellular tight junctions that are localized at the apical surface, and are pivotal for epithelial adhesion and barrier function.^8^ These tight junctions ensure the cells adhere together to form a regulated impermeable barrier. This intracellular adhesion complex consists of interconnections of proteins and receptors including, zonular occluden (ZO)‐1, ‐2 and ‐3, occludins, claudins, and transmembrane junctional adhesion molecules. Tight junctions control paracellular permeability, and immediately below them are the adherens junctions, composed of β‐catenin and E‐cadherin, which mechanically connect the adjacent cells and initiate proliferation and differentiation.^8^, ^9^ The presence and function of these complexes can be influenced by cellular differentiation, exposure to a range of stimuli, such as allergens and pollutants, and may be altered in disease conditions, contributing to immunopathology.^10^, ^11^

## The lung microbiota in health and disease

The long‐held, but incorrect, theory of sterility of the lung outside of clinical infection means historically little work has evaluated the role of the lung microbiota in chronic lung disease. Advances in high‐throughput molecular sequencing technologies have debunked this notion, demonstrating that the epithelial surfaces of the human respiratory tract are colonized by a complex and dynamic microbiota, termed the ‘lung microbiome’, which plays a role both in health and disease.^14^ The most commonly used approach to the study of bacterial communities exploits high‐throughput sequencing of amplicons of the 16S rRNA gene, a highly conserved locus in the bacterial genome that contains specific variable regions that can be used for phylogenetic classification.^15^ Sequencing studies have demonstrated stark differences in the lung microbiome between health and disease, and it is becoming increasingly apparent that the lung microbiome may not only contribute to explain the pathogenesis of these diseases, but also serve as a prognostic marker and therapeutic target.^16^, ^17^

In health, the lung microbiota has a low density but harbours a prominent diversity of interacting microbiota. The airway microbiota in healthy lungs is dominated by the *Bacteroidetes*,* Firmicutes* and *Proteobacteria*. Prominent genera in the lower airways include *Prevotella*, *Veillonella* and *Streptococcus.* ^21^ The composition of the lung microbiome is determined by three factors: microbial immigration; microbial elimination; and the relative reproduction rates of its members. The main routes of microbial immigration to the lungs are inhalation of air‐borne bacteria, direct dispersion along mucosal surfaces and microaspiration.^14^, ^22^ The similarities in community composition between the oral and lung microbiota suggest that microaspiration is likely to be the dominant route of immigration.^14^, ^23^ Microbial elimination is an active process that is achieved through mucociliary clearance, cough and host immune defences.^24^ The environmental conditions necessary for microbial growth within the respiratory tract (e.g. pH, temperature, nutrient availability, oxygen tension and activation of host inflammatory cells) are heterogenous, and considerable regional variation can be observed in a single healthy lung. During lung disease, the balance between immigration and elimination is perturbed, leading to alterations in the lung microbiota, with bacteria exhibiting competitive advantages becoming predominant^14^ (Table 1). This overgrowth of bacterial species leads to a decrease in overall richness, and is associated with the progression of chronic lung diseases such as asthma, chronic obstructive pulmonary disease (COPD), cystic fibrosis (CF) and idiopathic pulmonary fibrosis (IPF).^16^, ^17^, ^19^, ^20^, ^25^ Furthermore, these studies have linked microbial dysbiosis with an increased morbidity and mortality in a multitude of chronic respiratory diseases.

**Table 1:** Summary of studies illustrating changes in the respiratory microbiome that occur during chronic lung disease

Disease		Taxonomic changes at the genus level^1^	Sample type	Cohort size	[Ref.]
Asthma	Increased abundance of *Haemophilus*		Bronchoscopy	11	16
	Increased abundance of *Haemophilus*, *Neisseria*, *Fusobacterium* and *Porphyromonas*		Bronchoscopy	42	29
	Increased abundance of *Streptococcus*		Nasopharyngeal	234	30
COPD	Increased abundance of *Lactobacillus*		Lung tissue	24	17
	Increased abundance of *Fusobacteria*, *Leptotrichia* and *Fusobacterium*		BAL	32	31
CF	Increased abundance of *Burkholderia*, *Streptococcus* and *Staphylococcus*		Sputum	23	25
	Increased abundance of *Pseudomonas*, *Staphylococcus*, *Stenotrophomonas* and *Achromobacter*		Sputum	17	32
	Increased abundance of *Staphylococcus*, *Streptococcus* and *Pseudomonas*		BAL	95	33
IPF	Increased abundance of *Haemophilus*, *Streptococcus*, *Neisseria* and *Veillonell*a		BAL	65	19
	Increased abundance of *Staphylococcus* and *Streptococcus*		BAL	55	20
	Increased abundance of *Streptococcus*, *Prevotella*, *Veillonella*, *Haemophilus* and *Pseudomonas*		BAL	35	26

BAL, bronchoalveolar lavage; CF, cystic fibrosis; COPD, chronic obstructive pulmonary disease; IPF, idiopathic pulmonary fibrosis.

^1^ Changes in the microbiota in cases compared with controls.

In addition to the bacterial component of the microbiome, there is evidence that other non‐bacterial organisms, including viruses and fungi, may play an important role in health and disease. Emerging evidence suggests that the lung virome and mycobiome have a significant impact on clinical outcome of chronic respiratory diseases such as asthma, COPD, CF and IPF.^27^, ^34^, ^35^ It is also increasingly recognized that respiratory viruses are a major cause of acute exacerbations of chronic pulmonary diseases, and that the host response to viral infection is dysregulated in these diseases. For example, studies support a mechanistic role for viruses in the initiation and progression of IPF. The human herpes virus (HHV) family, which includes cytomegalovirus, Epstein−Barr virus, HHV‐7 and HHV‐8, has received considerable attention as either an aetiological or exacerbating agent of IPF.^36^ In asthma, infection by respiratory viruses is a major trigger of wheezing in infants as well as exacerbations of asthma in older children. The infectious agents associated with these wheezing events include respiratory syncytial virus (RSV), human rhinovirus, parainfluenza, coronavirus and other viruses.^37^ Despite the fact that very little is known about the lung mycobiome compared with the bacterial microbiome, in recent years specific fungi have been identified in respiratory diseases.^38^, ^39^ It has also been shown that commensal fungi can affect the immune system and also modulate bacterial communities, thus contributing to the restoration of the bacterial microbiota following antibiotic treatment.^40^, ^41^ Although studies into the composition and function of lung viral and fungal communities are gaining momentum, research in this field is still in its infancy, and future mechanistic work is needed to yield novel insights into their role in the pathogenesis of pulmonary diseases.

## A homeostatic balance between the lung and its microbiota

Airway epithelial cells are in continuous contact with the environment, and this interaction is a crucial factor in maintaining stable homeostasis.^42^ The three essential components that contribute to the barrier function of airway epithelium are: mucociliary escalators that trap and remove inhaled microbes and noxious stimuli from the airways; intracellular tight and adherens junctions that regulate epithelial paracellular permeability; and secreted antimicrobial peptides that kill inhaled pathogens.^1^ The physiological features of the respiratory tract are pivotal for maintaining a balance between the lung and its microbiota. The pulmonary epithelium is composed of distinct cell types and is not continuous from the upper respiratory tract to the alveoli.^43^ In the large airways, the submucosal gland harbours the mucus and serous cells that are responsible for the production of most airway mucus.^44^ Towards the bronchioles, goblet and club cells produce mucus, whereas in the alveoli AEC1 and AEC2 secrete pulmonary surfactant.^45^, ^46^ Mucus is a gel composed mainly of water and glycosylated proteins, such as mucins.^48^ Mucins are the large glycoprotein constituents of airway mucus.^49^ MUC5AC from goblet cells and MUC5B from submucosal glands are the predominant gel‐forming mucins in the human airways.^50^, ^51^ Mucus is a major ecological niche for the microbiota; it promotes healthy interactions between microorganisms and epithelial cells through the formation of a thin mobile layer that is supported by the periciliary layer covering the cilia.^49^, ^52^ In health, the mucus layer provides an effective defence against epithelial injury; however, excessive mucus production is associated with increased susceptibility to viral infections, respiratory allergies, and contributes to obstruction in several respiratory diseases such as pneumonia, asthma, COPD and CF.^53^, ^54^ This obstruction hinders the ability of the cilia to transport mucus out of the lungs, and favours the selection of certain pathogenic bacteria that thrive in mucus and establish themselves in this obstructed niche.^55^ Furthermore, microaspiration is commonly observed in chronic lung disease, and gastro‐oesophageal reflux disease (GORD) has emerged as a frequent co‐morbidity in chronic respiratory disorders. Although GORD is typically confined to the lower oesophagus, in some individuals it can be associated with pulmonary microaspiration of gastric contents.^56^ The occurrence of aspiration can lead to increased levels of bile acid in the lungs, which can favour the establishment of microbial biofilms, composed of microbial cells that form structured communities embedded in an extracellular matrix.^57^ Bacterial and fungal biofilms have emerged as a mechanism of virulence for opportunistic pathogens including *Pseudomonas aeruginosa* and staphylococcal species.^58^, ^59^ A hallmark of biofilms is their remarkable antimicrobial tolerance, which allows them to persist in the host and evade the host immune system.^59^ Therefore, GORD‐derived bile and subsequent biofilm growth on host tissue may contribute to chronic respiratory infection. In addition, direct seeding of bacteria from the intestinal microbiota into the airways may play a role in influencing the interaction between the epithelium and the microbiome.^60^

The intracellular tight and adherens junctions provide fundamental adhesive contacts between neighbouring epithelial cells.^61^ Tight junctions are the main regulators of paracellular permeability, whereas the adherens junctions mechanically connect adjacent cells, and initiate the formation and maturation of cell−cell contacts.^8^, ^9^ During disease, barrier function is impaired leading to increased epithelial permeability and pathogen entry.^10^, ^11^ Upon infection, epithelial cells are able to respond by the release of humoral factors such as antimicrobial peptides that exert broad‐spectrum antimicrobial activity, and a number of these are upregulated in response to the microbial dysbiosis seen in disease.^62^ Defensins are the most abundant antimicrobial peptides, and comprise α‐defensin in neutrophils and β‐defensin in epithelial cells. Human β‐defensin 1, 2, 3 and 4 are largely expressed on airway epithelial cells and function in airway mucosal defence by targeting a wide range of respiratory pathogens. Their expression in airway epithelia may be constitutive or inducible by bacterial products or proinflammatory cytokines.^63^ Nutrient availability in each specific niche will also play a role in the maintenance and selection of the lung microbiota. Host compounds such as mucins, cytokines, defensins and lactoferrins are the nutrient source available for bacteria in the lungs.^1^, ^64^ Microbial growth rates can also be influenced by changes in oxygen tension. Within the lungs there is significant regional variation in oxygen tension that can alter population dynamics by affecting bacterial proliferation.^65^ In chronic pulmonary diseases, an increased mucus production promotes bacterial growth and leads to the formation of zones with low oxygen concentration and high temperature favouring the selection and maintenance of particular bacteria.^14^ Collectively, these differences in the cellular function and the physiological characteristics of the lungs (e.g. temperature, mucus, pulmonary surfactant and oxygen tension) have a major impact on the establishment and persistence of the bacterial communities.

## The airway epithelium as a sensor of microbial presence

The airway epithelium not only represents a structural barrier but is also integral for innate host defence. Airway epithelial cells express several pattern recognition receptors (PRRs), and secrete antimicrobial molecules and mucins that aid in the defence against invading pathogens.^3^ PRRs are germ‐line encoded receptors that recognize conserved pathogen‐associated molecular patterns (PAMPs) or cell damage‐associated molecular patterns.^66^, ^67^, ^68^ These transmembrane receptors can be activated independently of the adaptive immune response and are classified into four families: the toll‐like receptors (TLRs), the cytoplasmic proteins retinoic acid‐inducible gene‐I‐like receptors (RLRs), the NOD‐like receptors (NLRs) and the C‐type lectin receptors (CLRs).^69^

To date, 10 (TLR1−TLR10) have been identified in the human genome, and are characterized by N‐terminal leucine‐rich repeats, a transmembrane region and a cytoplasmic Toll/IL‐1 receptor (TIR) homology domain that mediates signalling via MyD88 and TIR‐domain‐containing adapter‐inducing interferon‐β (TRIF).^70^ Toll‐like receptors play an important role in pathogen recognition and innate immunity. The main function of TLRs in mammals is to recognize distinct PAMPs, including components of microbial cell walls (TLR1, TLR2, TLR4 and TLR6), flagellin (TLR5), single‐stranded or double‐stranded viral RNA (TLR3, TLR7 and TLR8) or CpG‐containing DNA (TLR9).^71^, ^72^, ^73^, ^74^ The TLR3, 7, 8 and 9 are specifically localized in endosomes and detect pathogen nucleic acids following endocytosis.^75^, ^76^ Lastly, TLR10 is expressed in humans, but the specific ligand for it remains to be elucidated.^80^ Signalling through TLRs is key in early inflammatory responses to bacteria through the activation and expression of host cytokines, chemokines and antimicrobial peptides. TLRs are localized on immune and inflammatory cells; however, given that airway epithelial cells come into contact with several potential pathogens, the expression of TLRs is also relevant to immunity in the airways. Indeed, human bronchial epithelial cells express most TLRs; TLR2, TLR3, TLR5 and TLR6 being the most highly expressed.^81^

The PRR system can be also activated in response to allergens and viruses.^82^, ^83^ PRRs within the epithelium are able to recognize allergen‐associated danger signals via protease‐dependent and protease‐independent pathways. Protease‐dependent pathways specifically require direct cleavage of cell‐surface molecules or the activation of G‐protein‐coupled protease‐activated receptors (PARs).^85^, ^86^, ^87^, ^88^ RLRs are cytoplasmic receptors that recognize the presence of genomic RNA or double‐stranded RNA viruses or double‐stranded RNA intermediates from single‐stranded RNA viruses. Melanoma differentiation‐associated gene‐5 is expressed in human bronchial epithelial cells, and is involved in the detection of rhinovirus and other viruses.^89^ NLRs are intracellular PRRs characterized by a central nucleotide‐binding and oligomerization domain (NOD) and C‐terminal leucine‐rich repeats.^90^ More work is needed to define the role of NOD function in airway epithelial cells. Although the contribution of epithelial cells to antibacterial and antiviral immunity is well established, there is a paucity of information regarding the role of airway epithelial cells in immunity to fungi. CLRs belong to the dectin family and recognize fungal cell wall components. For example, Dectin‐1 is expressed in airway epithelial cells, and is responsible for their activation by mycobacterium, *Aspergillus* and allergens such as house dust mite.^91^, ^92^ Dectin receptors can be activated by β‐glucans via TLR4 signalling, TLR2‐dependant signalling, or induction of oxidative stress by intrinsic NADPH oxidase activity.^94^, ^95^ Because fungal spores are potent allergens and are associated with allergic and chronic diseases of the respiratory tract such as asthma, the importance of the lung mycobiome should not be overlooked.^97^

## Respiratory infections and the initiation of epithelial immune response

**Figure 2:**
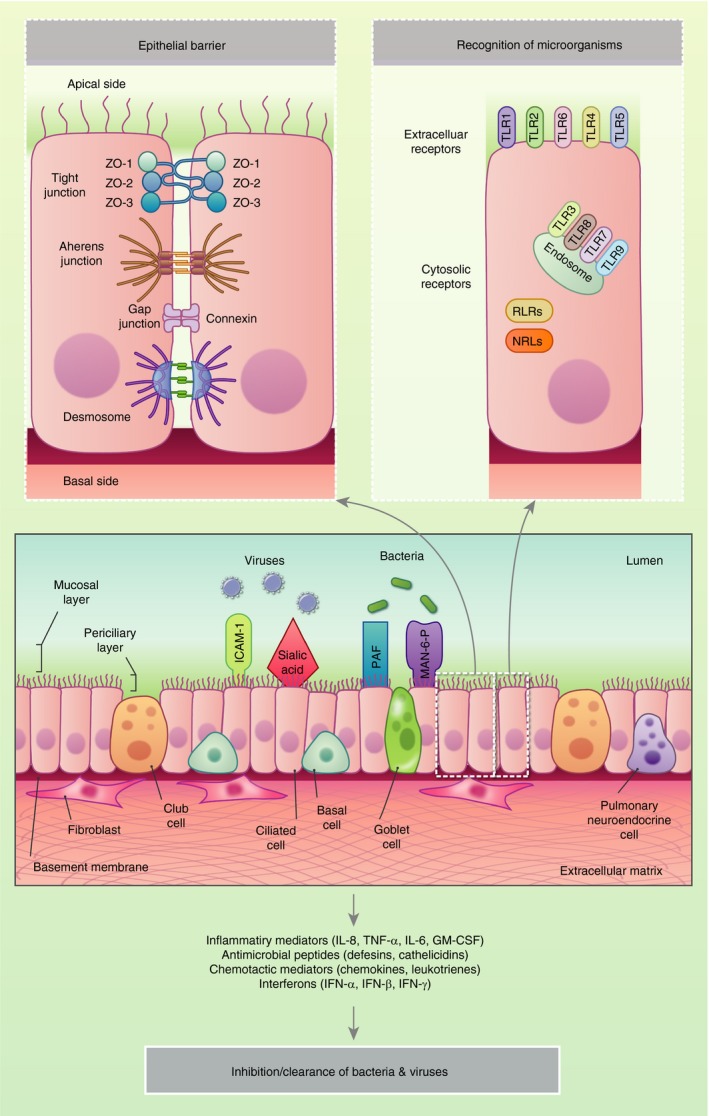
Response of the airway epithelium to infection. Bacteria and viruses bind to cellular receptors, including intercellular adhesion molecule 1 (ICAM‐1), sialic acid residues, platelet‐activating factor (PAF) and mannose‐6‐phosphate (Man‐6‐P). The pathogens are internalized in airway epithelial cells, which induces the production of innate immune defensins and the stimulation of intracellular and extracellular immune receptors such as the toll‐like receptors (TLR), retinoic acid‐inducible gene‐I‐like receptor (RLRs) and NOD‐like receptors (NLRs). In healthy individuals, pathogen clearance is achieved following the generation of pro‐inflammatory innate and adaptive immune responses.

Infections play a crucial role in the induction and exacerbation of a multitude of chronic respiratory diseases such as asthma and COPD.^98^ Such infections represent a major cause of morbidity and mortality globally, and may involve bacteria (e.g. *Streptococcus pneumoniae*, *Haemophilus influenzae, Chlamydia pneumoniae*) and viruses (e.g. RSV, influenza).^99^, ^100^ The initiation of infection typically occurs through the exposure of the respiratory tract to bacterial cells or viral particles (Fig. 2).

During infection, viruses can bind to specific cellular receptors such as intracellular adhesion molecule 1 (ICAM‐1) and sialic acid residues, whereas bacteria bind to platelet‐activating factor (PAF) and mannose‐6 phosphate (Man‐6‐P) receptors.^101^ The interaction between these infectious agents and the epithelial cells induces the stimulation of the PRRs, and the secretion of antimicrobial effector molecules, peptides, enzymes, reactive oxygen species together with a range of chemokines and growth factors. Collectively, these molecules facilitate the recruitment of and communication between immune cells, and contribute to the initiation of the innate immune responses that are pivotal for early control of an infection. In healthy individuals, this response promptly results in pathogen clearance. In contrast, in patients with chronic respiratory diseases these defences may be impaired leading to increased susceptibility to infection that drives inflammatory responses that in turn exacerbate the underlying disease. During chronic disease there is evidence that this barrier function is impaired, with disruption of tight junctions and increased epithelial permeability. This loss of barrier function leads to the entry of pathogens and other particles. Compromised barrier function is intimately linked to triggering of abnormal repair and remodelling.^102^ Respiratory viruses, air pollutants and allergens all have the capacity to inflict such damage on the epithelium, either directly due to inherent proteolytic activity or indirectly by binding to PAR‐2 expressed on the apical surface of bronchial epithelial cells. For example, *in vitro* studies have shown that infection of the cells with rhinovirus results in the loss of ZO‐1 from the junctional complex resulting in decreased transepithelial electrical resistance (TEER) indicative of barrier ‘leakiness’. Both coxsackie and rhinovirus affect occludins^103^, ^104^ whereas RSV causes disassembly of junctional complexes.^105^ In asthma, both decreases in ZO‐1 and TEER that correlate with asthma severity^106^ or E‐cadherin and β‐catenin have been shown *in vitro.* ^107^ Furthermore, reduced expression of occludins and claudins has been shown in COPD.^108^ *In vivo*, leakage of proteins such as albumin into the alveolar space has been reported following influenza infection.^109^ Viruses, pollutants and allergens are all associated with junction dysfunction and exacerbated clinical symptoms across the spectrum of these diseases.^103^, ^104^, ^105^

Defects in barrier structure and function have been observed in primary epithelial cell cultures from asthmatics even after being propagated for several weeks at air−liquid interface, suggesting that reduced barrier function is a stable property of these cells.^112^ The type 2 cytokines IL‐4 and 13 induce barrier dysfunction by inhibiting expression of ZO‐1, occludins, E‐cadherin and β‐catenin.^113^ It has been proposed that junctional disruption could be a T helper 2 (Th2) promoting signal in the airway to restore mucosal integrity.^114^ This idea is supported by the fact that siRNA knockdown of E‐cadherin results in epidermal growth factor receptor (EGFR)‐dependent production of Th2‐promoting cytokines thymus‐ and activation‐regulated chemokine (TARC) and thymic stromal lymphopoietin (TSLP) by airway epithelial cells that explains the association between barrier dysfunction and Th2‐driven pulmonary disease.^115^ If the wound healing response is not curtailed following restoration of mucosal integrity, detrimental airway remodelling can ensue. E‐cadherin, in addition to having a structural role at adherens junctions, is also the ligand for CD103, which is expressed on innate and adaptive immune cells and a subset of dendritic cells. Furthermore, it also binds to KLRG1, which is expressed by natural killer cells and regulatory T‐cells and group 2 innate lymphoid cells. E‐cadherin and occludin are known to be downregulated in asthmatics,^106^, ^116^, ^117^ and the genes for E‐cadherin and protocadherin‐1 have been associated with airway hyperresponsiveness.^118^, ^119^ E‐cadherin anchors EGFR within adherens junctions,^120^ preventing activation of the receptor.^121^, ^122^ EGFR can redistribute to the luminal surface of the cell as a result of reduced expression of E‐cadherin culminating in activation.^123^ Excessive activation of EGFR can result in cell proliferation and goblet cell hyperplasia/metaplasia.^124^ Increased expression of EGFR has been observed in asthma and correlated with impaired mucosal barrier function.^106^

When dissociated from E‐cadherin, β‐catenin can translocate to the nucleus, activating Wnt signalling, which results in cell proliferation.^125^ Dikkopf (DKK) proteins regulate Wnt signalling via interaction with Kreme (KRM) receptors,^126^ and the Wnt/β‐catenin pathway has been implicated in IPF.^127^, ^128^ DKK1 expression is increased in lung and bronchoalveolar lavage (BAL) fluid of IPF patients. In addition to being located in hyperplastic alveolar cells, the strongest expression is observed in basal bronchial epithelial cells. KRM1 expression is also elevated in the lung tissue of IPF patients, particularly in areas of bronchiolization.^127^ The phosphorylation signatures of β‐catenin in IPF are the same as in normal lung development, again suggesting that repair of lung injury involves reactivation of developmental programmes.^129^ Wnt/β‐catenin signalling is also implicated in the pathogenesis of COPD, but in direct contrast to what is observed in IPF, signalling in the mesenchymal compartment is inactivated rather than activated.^130^ Interestingly a shift from canonical Wnt signalling (β‐catenin‐dependent) to non‐canonical pathways is suggested to drive pathology in IPF.^131^

Basal cells, which are candidate stem cells in the conducting airways, are found in highest concentration in the trachea, and numbers diminish further down the bronchial tree to the bronchioles. Upon damage these cells upregulate p63, a basal cell‐restricted transcription factor, which has been implicated in epithelial mesenchymal transition.^132^ It has been shown that basal epithelial cells overlying fibroblastic foci in IPF acquire increased reactivity, determined by p63 expression, and gain a partial mesenchymal phenotype,^133^ further evidence of the involvement of bronchial‐derived cells in the pathogenesis of IPF.

Collectively, it is likely that the dialogue between specific bacterial communities and host is a dynamic phenomenon. The installation and location of the bacterial communities within the lungs is affected by the cellular components within the lungs as well as the abiotic environment (temperature, pH, mucus and pulmonary surfactant). Specifically, loss of barrier function can influence the composition of the respiratory microbiota by allowing the entry of pathogens and other particles. However, it is evident that more information is needed to elucidate the nature of the dialogue occurring between the respiratory microbiota and epithelial cells in the lungs to further understand the function and mechanisms through which the microbiota shapes immunity in the lung.

## The gut−lung axis

An increasing number of studies have provided evidence of the cross‐talk that occurs between the intestinal microbiota and the lungs, termed the ‘gut−lung axis’. Similar to the gut, the airway microbiota in healthy lungs is dominated by the short‐chain fatty acid (SCFA)‐producing bacteria: *Bacteroidetes*, *Firmicutes* and *Proteobacteria*. These are metabolically active bacteria that produce SCFAs via the bacterial fermentation of non‐digestible carbohydrates by the gut microbiota; acetate, propionate and butyrate being the most abundant in the human intestinal tract.^134^ It is now well documented that the intestinal microbiota influences the microbial composition within the lung either by direct seeding of bacteria into the airways or by the distribution of SCFAs. These SCFAs not only act as a fuel for intestinal epithelial cells but also exert distinct physiological effects, acting as a link between the microbiota and the immune system, and playing a key role in maintaining mucosal immunity. Although there are limited studies investigating the impact of these SCFAs in the lungs, in the gut they have been shown to be integral for tight junction formation and to promote anti‐inflammatory mechanisms to sustain intestinal homeostasis.^135^ Indeed, SCFAs interact with immune cells, and modulate their recruitment, differentiation, activation and survival at different tissues.^136^ The proposed pathway of this gut−lung interaction involves the entry of the microbiota and its products from the airways into the intestinal mucosa. These are phagocytosed and transferred to the mesenteric lymph nodes by antigen‐presenting cells that stimulate the priming of B‐ and T‐cells. Once activated, these cells can then migrate back to the original site or to distal locations such as the lung epithelium where they can exert directly on their target. Another proposed pathway involves the direct migration of bacterial products from the intestinal mucosa to the lungs via the bloodstream where they act to stimulate the immune system. Similarly, the same route is proposed beginning in the lung and culminating in the intestinal mucosa, although the influence of the lung microbiota and its metabolites on the gut is yet to be elucidated.^137^

The composition of a stable gut microbiota has been shown to have an influence on lung immunity. Mice devoid of their intestinal microbiota during early life are at an increased risk of developing allergic airway disease.^138^, ^139^ An increased risk of asthma has been associated with a predominance of *Bacteroidetes fragilis* and total anaerobes in early life,^140^ decreases in the total abundances of *Escherichia coli*, *Faecalibacterium*, *Lachnospira*, *Rothia* and *Veillonella* bacterial genera.^141^ In adults, taxa‐specific differences in the gut microbiota were observed, such as an increased relative abundance of *Bifidobacterium adolescentis*, which negatively correlated with the time since asthma diagnosis.^142^ Furthermore, mice fed a peptide‐based enteral diet that increased the production of SCFAs attenuated elastase‐induced inflammation and emphysema in the lung.^143^ The axis is by no means one‐directional, with respiratory infection driving perturbations in the gut microbiome, but further work is needed to validate this.^144^, ^145^ A study reported that the intranasal delivery of a non‐absorbable tracer into the nasal cavity of mice appeared in the gastrointestinal tract shortly after.^144^ In addition, work by Sze et al.^146^ reported that intratracheal administration of lipopolysaccharide not only disrupted the airway microbiota but also led to the translocation of airway bacteria belonging to the *Clostridium* and *Lachnospiraceae* genera into the bloodstream and affected the gut microbiota within 24 hr, resulting in an evident increase in total bacterial load. Collectively, these studies provide evidence of the bidirectional communication happening between these two sites. However, our understanding of this cross‐talk is still in its infancy, and there is a pressing need to uncover whether these changes observed in the microbiota are the cause or the effect of disease. Furthermore, there is growing evidence highlighting the use of oral antibiotics and their effect on lung immunity. Antibiotics could affect the lung microbiome either directly or indirectly through their effects on the gut microbiome and subsequently on lung immunity. To support this hypothesis, murine studies have shown that depletion of certain species within the gut microbiota due to antibiotic intake influences lung diseases and allergic inflammation.^147^ For example, microbial dysbiosis due to the use of macrolides in early life correlated with an increased risk of asthma in Finnish pre‐school children.^148^ It is clear that we need to appreciate the interactions between the gut and airway microbiota in order to understand the impact of antibiotics on lung health.

## Conclusions and future directions

The epithelial surfaces of the lung, including the proximal and distal airways, are now considered to play a pivotal role in maintenance of immune homeostasis. The epithelium acts as the first line of defence against inhaled viral, bacterial and fungal pathogens. Studies have shown that the epithelial surfaces of the human respiratory tract are colonized by a complex and dynamic microbiota that plays a role both in health and disease. It is becoming increasingly recognized that the cellular and physiological characteristics of the lungs impact the establishment and persistence of these bacterial communities. Dysregulation of the epithelial immune response and barrier function along with microbial dysbiosis can contribute to chronic inflammatory lung diseases such as asthma, COPD, CF and IPF. However, the precise nature of the relationship between the respiratory microbiome and the epithelium in the lungs remains an active area of investigation. A better understanding of this interaction in maintaining airway homeostasis will provide new insights into the pathogenesis of several respiratory conditions as well as novel diagnostic and therapeutic opportunities. Emerging evidence, particularly involving SCFAs, has highlighted a link between the gut and the lung and the microbial communities within. These results highlight the need for future studies that will characterize the microbiota and metabolites comprising these two mucosal sites and determine their interaction with the host in order to develop more targeted treatments for lung diseases.

## Disclosures

The authors have no competing interests or conflicts of interest to declare.
